# The memory effect of nanoscale memristors investigated by conducting scanning probe microscopy methods

**DOI:** 10.3762/bjnano.3.82

**Published:** 2012-11-06

**Authors:** César Moreno, Carmen Munuera, Xavier Obradors, Carmen Ocal

**Affiliations:** 1 International Center for Young Scientists, National Institute for Materials Science, Tsukuba, Ibaraki 305-0047, Japan; 2 Institut de Ciència de Materials de Barcelona, CSIC, Campus de la UAB, 08193 Bellaterra, Spain

**Keywords:** conductive scanning probe micoscopy, memristor, 3-D modes, resistive switching, scanning probe microscopy

## Abstract

We report on the use of scanning force microscopy as a versatile tool for the electrical characterization of nanoscale memristors fabricated on ultrathin La_0.7_Sr_0.3_MnO_3_ (LSMO) films. Combining conventional conductive imaging and nanoscale lithography, reversible switching between low-resistive (ON) and high-resistive (OFF) states was locally achieved by applying voltages within the range of a few volts. Retention times of several months were tested for both ON and OFF states. Spectroscopy modes were used to investigate the *I*–*V* characteristics of the different resistive states. This permitted the correlation of device rectification (reset) with the voltage employed to induce each particular state. Analytical simulations by using a nonlinear dopant drift within a memristor device explain the experimental *I–V* bipolar cycles.

## Introduction

The current knowledge-based society requires a new, more-powerful memory technology for the development of any field concerning human activity, such as biomedicine, space research, meteorological predictions, simulation in basic research science, and entertainment. Nowadays computers use two types of memory, i.e., dynamic random-access memory (DRAM) and static random-access memory (SRAM). The handicap of these fast memories is that they are volatile, i.e., data are lost when the power supply is removed. For this reason computers also use a nonvolatile memory, i.e., hard-disk drives (HDDs). HDDs are nonvolatile but also slow, thus increasing the computer start-up time. Another kind of nonvolatile memory commonly employed for data storage in hard disks, digital cameras, USB memory sticks, or cellular disks is Flash memory. Its main drawbacks are a slow writing speed and the limited number of write/erase cycles that can be endured.

At this point, the need for new materials with higher performance has encouraged extensive basic research in this field. Resistive random-access memory (RRAM) based on a memristive behaviour has become an exciting and strongly developing field, because when combined with transistors in a hybrid chip, memristors could radically improve the performance of digital circuits without the need for further reduction of transistor dimensions [1]. These memory resistors are similar to nonlinear resistors exhibiting memory effects and are essentially two-terminal devices whose resistance depends on the polarity of the applied voltage. The simplest memristor consists of a thin oxide or semiconductor doped film (of thickness *w*) between top and bottom metallic electrodes. The slope of the functional relationship between the time integral of the current and the time integral of the voltage across the device element is the so-called memristance, *M*(*w*), similar to a variable resistance, which provides a pinched effect to the characteristics of the devices.

Memristance becomes more important as the critical dimensions shrink to the nanometre scale [2] and therefore the characterization of the local electrical properties becomes more and more important. In this work we have combined conductive scanning force microscopy imaging and single-point current–voltage spectroscopy, with more advanced spectroscopy measurements (3-D modes) to characterize the nanoscale electrical response of thin films presenting memristive behaviour. The systems of choice for benchmarking are ultrathin films of conducting La_0.7_Sr_0.3_MnO_3_ (LSMO) with a thickness in the range of 10 to 24 nm, grown by chemical solution deposition (CSD). The films are transferred through air to the SFM equipment, and therefore, a thin top layer likely with a slightly different oxygen content is expected to cover the conducting LSMO film, which otherwise acts as the bottom electrode. The present work particularly aims at demonstrating and evaluating the electrical responses, including the memory effect, of nanoscale memristors, by a combination of contact C-SFM modes, which reveal the technique as an ideal tool for the research and development of memristive systems on a nanometre scale.

## Results and Discussion

### Resistive-switching procedure

Conductive scanning force microscopy was used either to modify (writing/erasing) or to characterize (reading) the electrical properties of the sample under study. Current images were acquired in a noninvasive manner (no sample indentation) by using the contact operation mode at the lowest possible applied load while still obtaining stable signals. The conducting tip (top electrode) was placed in direct contact with the sample surface, under controlled load, i.e., by using a normal force feedback, and the current between the tip and sample was measured. Simultaneous topographic images *z*(*x*,*y*) and current maps *I*(*x*,*y*) over a given region at a fixed voltage permitted observation of the complete writing (positives *V*_tip_), reading (small negative *V*_tip_) and erasing (negatives *V*_tip_) process. In our setup, the sample was always grounded and the voltage was applied to the tip.

Given the insulating character of the substrates used (SrTiO_3_), the direct electrical contact to ground was established through a metallic clamp (counter electrode) firmly attached to the film at the sample border (millimetres apart from the tip–surface contact). For topographic images a colour code is commonly used in which bright colours indicate high values and dark means low; however, interpretation of colour-coded current maps depends on the voltage sign as well as the absolute current magnitude. Thus higher currents appear darker in C-SFM images taken at negative *V*_tip_, while brighter for positive *V*_tip_. The current–voltage (*I–V*) characteristics of the contact were measured as a function of the bias voltage applied between tip and sample, starting from negative tip voltages.

**Figure 1:**
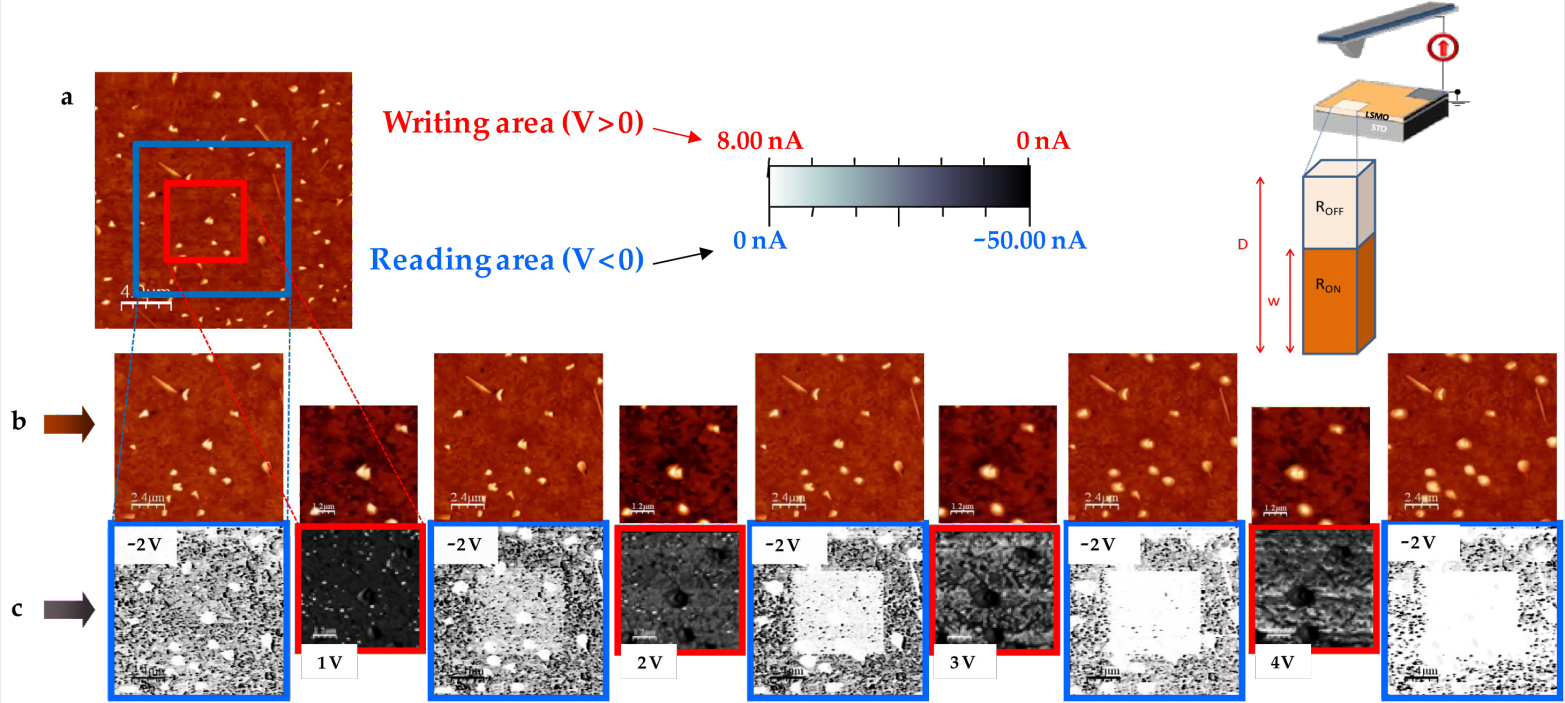
Resistance-switching sequence: Writing and reading of local conductance modifications made on the LSMO surface. The transition between low-resistive (LR) and high-resistive (HR) states is performed by writing at an appropriate *V*_tip_ with the tip of the conducting scanning force microscope (C-SFM). (a) Topographic image (20 μm × 20 μm) of a 24 nm thick LSMO thin film. A certain concentration of outcropped insulating (La,Sr)*_x_*O*_y_* (LSO) nanodots (50–80 nm high) are used as an in situ reference for topographic and conductivity measurements. Concentric squares on the image correspond to the writing (inner square) and reading (outer square) regions. Top-right inset: Schematics of the measuring setup indicating relevant parameters. (b), (c) Series of topographic and current-map images displaying the reading area (12 μm × 12 μm) taken at *V*_rd_ = −2 V, before (virgin state) and after writing a HR area (6 μm × 6 μm) in several steps *V*_wr_ = +1, +2, +3, +4 V. Note: the colour code in the current maps depends on the voltage sign, and therefore a dark colour means higher current for *V*_tip_ < 0 but lower current for *V*_tip_ > 0 (see scale bar).

Once the surface location to be modified had been selected by large-scale topographic imaging, typical writing–reading C-SFM experiments, as presented in Figure 1, were based on the following protocol:

1. The scan range is set to the reading area. The tip is biased at *V*_rd_, and topography and current images are simultaneously acquired to characterize the conductance state of the surface.
2. The scan range is reduced to the writing area. The *V*_tip_ is set to a given positive value, and topography and current images are simultaneously acquired.
3. The *V*_tip_ is brought back to the reading value (*V*_rd_) and any possible modification is checked by repeating (i). The process in (ii) is repeated at increased *V*_tip_ until no current is detected within the writing region. The corresponding voltage, which will be seen to depend slightly on film thickness, is considered as the onset for writing, *V*_wr_.

**Figure 2:**
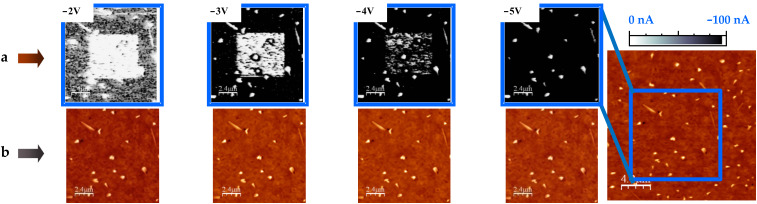
Resistance-switching sequence: Erasing back to a LR state starting from the local HR (nonconducting) modification, performed on the LSMO surface at *V*_wr_ = +4 V. The nonconducting character of the written region (white square) is reversed by changing the voltage polarity: (a) Reading process at *V*_rd_ = −2V and three steps of the erasing process *V*_er_ = −3, −4 and −5 V. The conducting character of the initial LSMO is recovered after applying *V*_er_ ≈ −*V*_wr_. (b) Series of topographic images of the erased region show that the process does not result in morphological changes.

The retention time of the written high-resistive state of Figure 1 is further investigated in the following sections. Nonetheless, as illustrated in Figure 2, if desired, a total or partial switch back to the conductive state can be accomplished by scanning the modified region (or a portion of it) at a negative *V*_tip_ for which the conducting character is recovered. This is called the erasing voltage, *V*_er_, and has been found to be at least *V*_er_ = −*V*_wr_ [3]. The reversible transition between differently resistive states of the LSMO thin film are performed (writing and erasing) and followed (reading) in a noninvasive way.

### Analytical simulation of the memristor *I–V* behaviour

**Figure 3:**
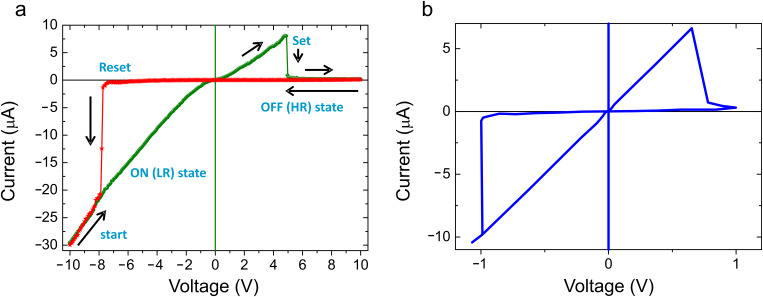
Experimental (a) and simulated (b) *I–V* curves. (a) Starting from a conducting (LR) state, a voltage ramp is applied to the tip, from negative to positive values and back, to complete the cycles as indicated by the arrows. *Set* indicates the change from the initial conductive state to a more resistive one and *Reset* corresponds to the restoration of the ohmic behaviour. LR and HR states are identified as ON and OFF, indicating the pass or blockage of electric current through the device. To have access to the complete range of currents (1 pA to 1 mA) an external *I–V* converter was used (see Experimental section). (b) Calculated *I–V* curve for a memristor with a nonlinear dopant drift under an external sinusoidal bias with a voltage amplitude *v*_0_ = 10 V, a resistance ratio *R*_OFF_/*R*_ON_ = 70 and a memristor length *D* = 10 nm. The units of the axes are expressed in units of voltage amplitude *v*_0_ = 10 V and the unit of current is *I*_0_ = *v*_0_/*R*_ON_ = 10 μA. *I*_0_ is the maximum current reached through the memristor.

Typical *I–V* characteristics conducted by application of a voltage cycle at a fixed location on the virgin (preswitched) 10 nm thick LSMO thin film surface presented a bipolar switching behaviour, such as that shown in Figure 3a. The clearly asymmetric curve indicates that diverse microscopic processes govern charge transport under each polarity; the different branches are capable of being described by *I–V*s of electrical circuits in which both resistors and rectifiers are involved, as already observed in other thin-oxide-based memristor systems [4].

The fundamental memristive system theory states that the observation of a pinched current-versus-voltage hysteresis loop measured from an experimental two-terminal device, when driven by a dc and/or sinusoidal signal of any frequency, implies that the device is a memristor [5]. A basic mathematical definition of a memristor, assuming an ohmic electron conduction and linear ionic drift in a uniform field, is given by the following equations [2]:

[1]
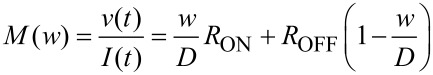


[2]
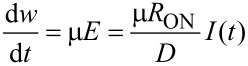


where *M* is the memristance, *w* is the size of the doped region, *D* is the thickness of the film, *R*_ON_ and *R*_OFF_ correspond to the resistance of the memristor for completely uniformly doped or undoped cases, respectively, and µ is the drift velocity of the dopants under an electric field *E* across the doped region in the presence of a current *I*(*t*).

The switching characteristic observed for a particular memristive system helps to classify the nature of the dopant drift inside the memristor. If the generated electric field can be assumed to be small, the linear dopant-drift model can approximate the dynamics of a memristor. However, this model is not valid in our case. During the electrical measurement of the *I–V* characteristic in contact mode, an enormous electric field can be generated considering the small tip–sample contact area and the Schottky-like tip–film contact. Numerical simulation for 60 nm thick films shows that the electric field within a few nanometres from the tip–sample contact is a factor of ~20 larger than that in region of the film closer to the substrate interface [6].

**Figure 4:**
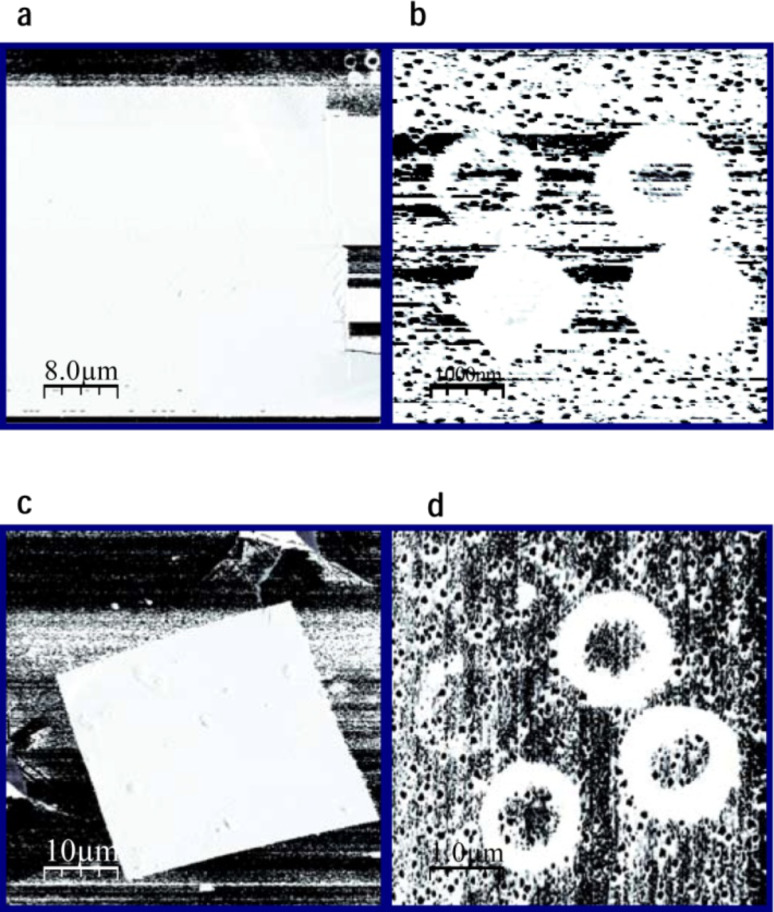
Current-map reading images of different high-resistive modifications. As-fabricated nonconducting square region (a) and a series of HR rings written at different increasing *V*_wr_ from left to right and top to bottom (b). The bottom images (c and d) were taken after four months by relocating the samples in the C-SFM system to observe the same surface regions (slightly rotated). The samples were annealed at up to 400 K and submitted to magnetic fields (up to 3 T) as part of endurance tests between sets of images. The images were taken at *V*_rd_ = −2 V and the total scale goes from 0 to −100 nA (white to black).

The influence of a nonuniform electric field significantly suppresses the drift of the dopants. Nonlinear dopant drift can be taken into account in the memristor simulation *I–V* curve by introducing an appropriate window function in Equation 2. Thus, by using the window function proposed by Joglekar [7], i.e.,

[3]
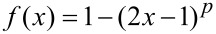


with *p* being a positive exponent , we obtain the simulated *I–V* curve showed in Figure 3b, which corresponds to a memristor in a nonlinear model in which the dopant drift is heavily suppressed only near the boundaries (*p* = 10). Experimental and simulated curves are in excellent agreement, pointing out that the measured hysteretic *I–V* curve is consistent with a hard-switching system governed by nonlinear ion drift in a high electric field. These nonlinearities have been related to long retention times and fast switching processes [8] in accordance with the endurance test displayed in Figure 4.

In order to gain further insight into the nature of the process, the durability of the tip-voltage-induced modifications was tested as a function of time, temperature and magnetic field. As can be seen in the current images presented in Figure 4 for different geometries of the modifications (square and rings) and different *V*_wr_, the initial high-resistive states (a and b) were found to persist for at least up to four months, as well as upon annealing up to 400 K, and show no important degradation under magnetic fields up to 3 T (c and d). The magnetic-field independence is what is to be expected from the memristor equations. Note that, as well as the square-shape modification (a, c), the HR rings persist in all cases after this long period of time, excluding important degradation. The small differences in the measured current between b and d are ascribed to slightly different reading conditions. However, a thin contamination layer adsorbed during exposure to air and the endurance treatments, connecting the inner ring with the outer conducting region, cannot be excluded. A certain contribution arising from these effects would be similar for all modifications, independently of the initial writing parameters; therefore, a comparative spectroscopic investigation is presented in the following to obtain direct comparison between electric-current behaviour for different HR states.

### Complementary memory tests: 3-D modes and *I–V* spectroscopy

**Figure 5:**
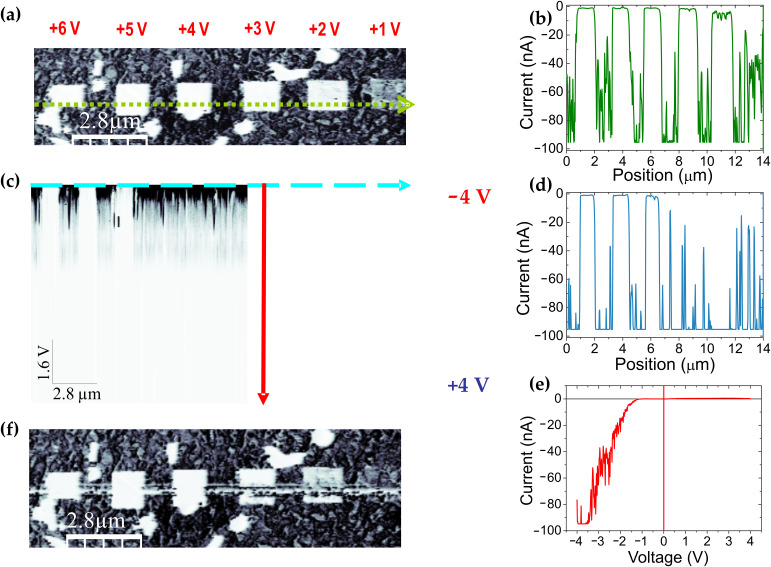
3-D mode spectroscopy. (a) Current-map reading image (*V*_rd_ = −2 V) of different HR states written at *V*_wr_ from +1 to +6 V. (b) Current profile along the dotted green line marked in (a) (*V*_tip_ = −2 V). (c) *I*(*x*,*V*) image obtained at the same surface line: the colour scale corresponds to the current measured as a function of the lateral position (horizontal axis) and the voltage (slow scan) ranging from *V*_tip_ = −4 V (top) to +4 V (bottom). (d) Current profile along the horizontal blue dashed line in (c) (*V*_tip_ = −4 V) and (e) *I–V* curve corresponding to the vertical profile (red line in c) centred in the modification made at *V*_wr_ = +3 V. (f) Current-map reading image (*V*_rd_ = −2 V) after 3-D image. Because the voltage range (±4 V) of the 3-D image is high enough to modify the surface, a line has been written (LR to HR) between modifications at the tip passage. For a similar reason a line has been erased (HR to LR) at those modifications where the corresponding *V*_er_ has been exceeded. Note: the maximum currents measured (±100 nA) correspond to the saturation of the amplifier setup.

Figure 5a depicts a reading current map, *I*(*x*,*y*), performed over a surface region containing a series of squared HR regions obtained by writing at *V*_tip_ voltages ranging from *V*_wr_ = +1 to +6 V. As seen in the line profile *I*(*x*) of Figure 5b, taken along the green dotted line in Figure 5a, at the employed *V*_rd_ = −2 V the current approaches zero for all modifications performed with *V*_wr_ > +1 V. The maximum current measured (±100 nA) corresponds to the saturation of the current amplifier in the experimental setup used.

To analyse the memory effect of the fabricated memristors further in terms of the electronic current passing through different devices and the restoring voltages, a complementary C-SFM method is further proposed. This method is based on the so-called 3-D modes [9–10] and is used here to measure the current (*I*) as a function of the applied voltage (*V* = *V*_tip_) along a single surface line (*x*), thus obtaining a *I*(*x*,*V*) image in which the colour scale is the current measured at each pixel, the fast scan continuously sweeps the same surface line, and the voltage is changed at every line of the slow scan. This was the procedure used to obtain the 3-D image of Figure 5c: the tip was positioned to cross the whole series of modifications at the same green dotted line of Figure 5a and *V*_tip_ was changed from −4 to +4 V (fast scan). In such a way horizontal profiles in the image of Figure 5c provide the current values along the surface line at a given voltage (Figure 5d) and vertical profiles correspond to the characteristic *I–V* curve at a given point (*x*,*y*) (Figure 5e). As a result, the whole set of *I–V* curves were simultaneously obtained for an ensemble of surface locations lying along the selected line.

The first noticeable piece of information is extracted from the current profile taken at *V*_tip_ = −4 V and depicted in Figure 5d. Note that the *I*(*x*) measured on modifications made at *V*_wr_ = +2 and +3 V has considerably increased from that shown in Figure 5b (*V*_tip_ = −2 V). It is worth noting that as commented above the resistive switching is reversed for *V*_er_ > −*V*_wr_ and therefore, this observation is the result of a reversible switch (erasing process) from each HR to a more conducting LR state. Conversely, as expected HR states written at +4, +5 and +6 V remained unaltered. The reading image of Figure 5f recorded after the 3-D imaging further illustrates the reversibility of the process. On the one hand, as a consequence of the scanning at −4 V a line has been erased (HR to LR) on top of the squares written at +3 and +2 V. On the other hand, note that the *V*_tip_ reached during the 3-D imaging goes up to +4 V, modifying the virgin film surface (LR to HR) between the pre-existing marks. From the above results one can think about the capability of the proposed process to fabricate wire-like modifications, which are promising for nanodevice applications, made by the sweeping action of the tip at different voltages. The longitudinal dimension of such wires is only limited by the total piezo scan range (up to hundreds of microns in common SFM systems) and the short dimension is limited by the contact area of the tip and, therefore, dependent on the tip radius. At present, lines as thin as about 50–100 nm can be performed, demonstrating the scalability of the resistive-switching effect to nanoscale devices. It is worth mentioning here that, by comparing Figure 5a and Figure 5f we can rule out any tip effects in *I–V* measurements due to tip contamination or coating wear. The nonmodified regions of the LSMO films serve as an in situ quality test of the tip performance. No measurable changes were observed in the current measured on these nonmodified regions after the whole series of experiments in Figure 5, ensuring the good electrical performance of the tip and the reliability of the measurements.

**Figure 6:**
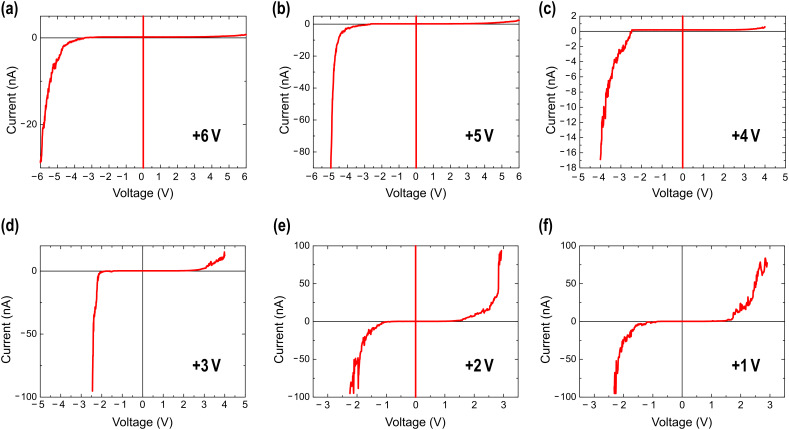
*I–V* curves obtained at specific surface locations within the different resistive modifications of Figure 5. The corresponding *V*_wr_ is indicated in each panel. The voltage range has been expanded to different values in order to visualize that rectification (reset) occurs at *V*_tip_ ≈ −*V*_wr_.

As will be confirmed below and has been previously demonstrated by means of Kelvin probe force microscopy [3], the results presented here support the memristor memory effect capable of memorizing the amount of charge that has passed through it. In order to gain insight to this issue, analysis of the *I–V* characteristics is needed. As commented above, vertical profiles in the *I*(*x*,*V*) would provide, in principle, such curves at each point of the selected line. An example of such *I–V* is shown in Figure 5e for a location within the modification performed at +3 V (vertical red line in Figure 5a). Though the curve is relatively noisy, it shows a clear rectifying behaviour. It is worth remarking that in the 3-D modes the current is measured during tip scanning, i.e., the point at which the tip contacts the surface is continuously changing, and therefore, some averaging and deviation of the true *I–V* behaviour can exist. In addition, as has been described above, the resistive state of a given modification is altered if the total range exceeds the absolute value of the corresponding *V*_wr_. Therefore, in order to explore the true *I–V* curve by improving the signal-to-noise ratio and using an adequate voltage range for each HR state, we also measured individual *I–V* curves, such as those presented in Figure 6.

*I–V* curves (Figure 6e and Figure 6f) obtained on the modifications made at the lower voltages (*V*_wr_ = +1 and +2 V) are nearly symmetric, with a pseudo-gap around *V* = 0. Interestingly, all *I–V* curves for larger *V*_wr_ (Figure 6a to Figure 6d) show a clear rectifying behaviour such that charge injection occurs exactly at *V*_Reset_ = −*V*_wr_ indicating that to change the memory state of the device, carriers must overcome a barrier height equal to the switch-on voltage employed in each case. At this point, the particular HR state is reversed to the LR state. In other words, *V*_Reset_ = *V*_er_. As already proposed for other memristive devices showing similar behaviour [4], a combination of a non-ohmic contact interface (maybe Schottky-like) and quasi-ohmic contact at the opposite interface could be responsible for the observed features in the electrical responses. This interpretation is in agreement with the nonuniform-dopant-drift analysis of the hysteretic characteristic cycles. Due to air exposure, the oxygen content of the topmost surface region of the LSMO may be different to the stoichiometric composition constituting the active doped element of the memristor.

As an alternative to other investigations made on memristor devices with two planar electrodes in which the active regions are buried under the metal contact, here we have been able to analyse the memory effect of nanoscale resistive switching with the very same probe that was used for the fabrication, providing direct evidence for the restoring phenomena as an activated process involving carrier injection. Integration of the presented nanoscale memristor devices opens new possibilities for the low cost and scalable development of ultrahigh-density resistive and nonvolatile memory cells.

## Conclusion

In this work we have shown the full capabilities of electrical scanning probe microscopy modes to modify and characterize memristive LSMO thin films on the nanoscale. Hard switching with long retention times and fast switching process are consistent with the nonuniform-drift model used here. We have proposed a nanoscale methodology to fabricate and characterize durable memristors using the tip of the C-SFM as a movable top electrode, as an alternative to standard two-plate devices.

The superb control of the top electrode and perfect interface between the native oxide and the bottom conducting LSMO electrode make the electrical characteristics of the memristor highly reproducible.

## Experimental

### Sample preparation

La_0.7_Sr_0.3_MnO_3_ (LSMO) films with a thickness in the range of 10–24 nm were grown by chemical solution deposition (CSD) on (100)SrTiO_3_ (STO) substrates [11]. The metal–organic solution was deposited by spin coating, at 6000 rpm for 2 min, and annealed at temperatures in the range of 900–1000 °C under an oxygen gas flux, for different times up to 12 h. Heteroepitaxial growth of LSMO films was confirmed by θ–2θ XRD patterns and XRD *q*-plot measurements showed that the films are fully strained. The values of resistivity, magnetoresistance and Curie temperature are very similar to those observed in LSMO films grown by physical vapour-deposition techniques, such as sputtering or pulsed-laser deposition, leading us to conclude that a similar epitaxial quality is achieved with films grown by our CSD approach. More experimental details of the growth process, as well as structural and magnetic characterization, have been reported elsewhere [12–13].

Between the retention and endurance tests, the samples were always stored at ambient conditions in a plastic box supplied with silica powders.

### Scanning force microscopy

Scanning force microscopy (SFM) measurements were performed using a commercial head and software from Nanotec [14] under a N_2_ environment (RH < 5%) to diminish any possible humidity effects. For the conductivity measurements (C-SFM), we used either commercial conductive B-doped diamond-coated tips with *k* = 2.8 N/m (Nanosensors) or Cr–Pt coated Si tips with *k* = 3 N/m, resonance frequency of 75 kHz and contact resistance of 300 Ω on a Pt thin film surface (Budgetsensors in-factory specifications). The same tip was used in all the C-SFM experiments of at least one series. To check tip–sample conditions, the adhesion force was systematically determined from force-versus-distance curves prior to and after each conductivity experiment. In addition, comparison of the conducting properties of nonmodified regions prior to and after the experiments was used as an in situ quality test to assess proper tip performance. Applied forces were commonly in the 50–100 nN range. For all measurements in this study, electrical testing was performed by applying the bias voltage to the top electrode (tip) keeping the bottom electrode (LSMO film) grounded. When needed, an external *I–V* converter (Stanford Systems) was used to gain access to a wide range of compliance currents (1 pA to 1 mA).
